# Prevalence rate and risk factors of human cystic echinococcosis: A cross-sectional, community-based, abdominal ultrasound study in rural and urban north-central Chile

**DOI:** 10.1371/journal.pntd.0010280

**Published:** 2022-03-09

**Authors:** Gerardo Acosta-Jamett, Felipe A. Hernández, Natalia Castro, Francesca Tamarozzi, Leonardo Uchiumi, Juan Carlos Salvitti, Michelle Cueva, Adriano Casulli

**Affiliations:** 1 Instituto de Medicina Preventiva Veterinaria, Facultad de Ciencias Veterinarias, Universidad Austral de Chile, Valdivia, Los Ríos region, Chile; 2 Center for Surveillance and Evolution of Infectious Diseases, Universidad Austral de Chile, Valdivia, Los Ríos region, Chile; 3 WHO Collaborating Centre for the Epidemiology, Detection and Control of Cystic and Alveolar Echinococcosis, Department of Infectious Diseases, Istituto Superiore Di Sanità, Rome, Italy; 4 Ramon Carrillo Hospital, Bariloche, Río Negro Province, Argentina; 5 Artémides Zatti Hospital, Viedma, Río Negro Province, Argentina; 6 European Reference Laboratory for Parasites, Department of Infectious Diseases, Istituto Superiore Di Sanità, Rome, Italy; Seoul National University College of Medicine, REPUBLIC OF KOREA

## Abstract

**Background:**

Cystic echinococcosis (CE) caused by *Echinococcus granulosus* sensu lato (s.l.) is a neglected and underdiagnosed parasitic zoonosis that has a significant socioeconomic impact on rural communities relying on livestock farming. CE is endemic across Latin America, including Chile, where the Coquimbo region exhibits a relatively high record of hospital-based human cases and infected animals. However, the incidence of hospitalized CE cases may underestimate the real burden of infection in a population, since the majority of cases never reach medical attention or official disease records.

**Methodology/Principal findings:**

In 2019, a cross-sectional, community-based study was conducted with the objectives of estimating for the first time the prevalence of human abdominal CE using abdominal ultrasound (US) screening in volunteers residing in urban and rural localities of the Monte Patria municipality located in Limarí province, Coquimbo region, Chile, and identifying the risk factors associated with human infection. Pre-screening activities included a 16-h lecture/hands-on training aimed at rural physicians that focused on the diagnosis of CE by US, based on current WHO recommendations. A total of 2,439 (~8% of municipality inhabitants) people from thirteen target localities were screened by abdominal US in June-July 2019. We found an overall CE prevalence of 1.6% (95% CI 1.1–2.2) with a significantly higher likelihood of infection in rural localities, older age classes and people drinking non-potable water; 84.6% of infected volunteers were newly diagnosed with CE. Cysts were either in active or inactive stages in equal proportions; active cysts were detected in all age classes, while 95.7% of inactive cysts occurred in >40 years-old subjects.

**Conclusions/Significance:**

This is the first US survey aimed at detecting human infection caused by *Echinococcus granulosus* s.l. in Chile. Our findings indicate a high CE prevalence in the area, and contribute to define the demographic and behavioral risk factors promoting the transmission of the parasitic infection within target communities. Our results support the implementation of cost-effective strategies for the diagnosis, treatment and control of CE, and the need to improve the epidemiological surveillance system in Chile.

## Author summary

Human cystic echinococcosis (CE) is a parasitic disease caused by *Echinococcus granulosus* s.l. complex; its cycle involves canids and livestock, mainly sheep. It is endemic in areas with extensive livestock breeding activities, where close contact occurs between humans and the domestic dog-livestock parasite cycle. After ingestion of parasite eggs, people may develop fluid-filled cysts, mainly in the liver. CE is a neglected disease, which causes significant socioeconomic impact mainly on poor communities worldwide. Prevalence data in many affected areas is lacking or largely underestimated, partly because human cases typically do not reach medical attention or official disease records. We determined the prevalence of abdominal CE by ultrasound population-based screening in volunteers residing in urban and rural localities of the Monte Patria Municipality, Limarí province, Coquimbo region, which is an *E*. *granulosus* s.l. endemic area of northern Chile, and assessed the risk factors associated with human infection. We performed an ultrasound survey on 2,439 people residing in thirteen localities and found an overall prevalence of 1.6% (95% CI 1.1–2.2). A higher risk of infection was associated with residing in rural localities, older age and drinking non-potable water. CE cysts were either in active or inactive stages in equal proportions and 84.6% of infected volunteers were newly diagnosed with CE. Our findings will serve to support the implementation of cost-effective diagnostic, treatment and control strategies and to improve the epidemiological surveillance system for CE in Chile.

## 1. Introduction

Cystic echinococcosis (CE) is a parasitic zoonosis caused by infection with the larval stage of *Echinococcus granulosus* sensu lato (s.l.) complex. The life cycle of these parasites includes most commonly domestic dogs (but also wild canids) as definitive hosts developing the adult tapeworm in the intestine while sheep and other ungulates (e.g. cattle, goats, pigs) act as intermediate hosts, where the larval stage develops as fluid-filled cysts in the viscera [1–3]. Dogs acquire the parasite by ingesting raw offal of intermediate hosts containing infective echinococcal cysts, accessed mainly during domestic or non-regulated livestock slaughtering [1–3].

Humans are accidental dead-end hosts for *E*. *granulosus* s.l., acquiring the infection through ingestion of viable parasite eggs released in the environment through the feces of infected dogs. In humans, CE cysts mainly develop in the liver, followed by the lungs, and secondarily other organs and tissues [4]. The course of infection is slow, and most infected individuals remain either asymptomatic for years or exhibit non-specific symptoms conducting to accidental diagnosis [4]. Parasite eggs can remain viable from several months up to a few years in the environment and diverse matrices [5]; consumption of contaminated food and water and direct contact with dogs are generally considered the most likely transmission routes of infection for humans [6,7]. However, the results of several studies on this topic are not univocal, and there is still a lack of information on actual matrices contamination, specific risk factors for eggs ingestion by humans, and increased odds of infection in specific urban and rural areas [8].

In Latin American countries such as Argentina, Brazil, Chile, Peru and Uruguay, CE is considered an important public health issue in pastoral communities [9,10]. In Chile, human CE is a notifiable endemic disease and the reported surgical incidence has remained stable at around 2.5 cases per 100,000 inhabitants since the early 1990s [11,12]. Official records from medical notifications have shown higher surgical incidence in southern regions (>30 cases per 100,000 inhabitants in Los Lagos, Aysén and Magallanes) when compared to northern regions (10–30 cases per 100,000 inhabitants in Coquimbo, Maule and La Araucanía) [11–14]. In the Coquimbo region, the highest surgical incidence of human CE in 1995–2006 was reported in the Limarí province, reaching 8.5 cases per 100,000 people [15]. In 2010–2012, the mean surgical incidence in the region was 2.6 cases per 100,000 inhabitants, reaching 21.3 cases per 100,000 inhabitants in the municipality of Monte Patria [16]. Common practices of animal management (e.g. keeping and having contact with dogs and their feces, and home-slaughtering of livestock) favor the transmission of the parasite in both rural and urban areas [17,18]. However, notifications of hospital cases may underestimate the real burden of disease, since the majority of infections remain asymptomatic for a long time or lifelong and, even when symptoms occur, the infection may be misdiagnosed or never reach medical attention or official disease records. Therefore, population-based studies complement hospital records and allow a better estimate of the occurrence and distribution of infections in a particular area [19–22].

In 2019, the Universidad Austral de Chile in partnership with the Italian Istituto Superiore di Sanità (project coordinator), participated in the collaborative, multicenter study entitled “Molecular-epidemiological studies on pathways of transmission and long-lasting capacity building to prevent cystic echinococcosis” (PERITAS 2019–2022; https://www.iss.it/en/web/iss-en/who-cc-peritas), funded by the European Commission under the umbrella of EU-LAC Health initiative. Other project’s partners included the Consejo Superior de Investigaciones Científicas (Spain), the Instituto de Salud Carlos III (Spain), the Universidad Nacional de Río Negro (Argentina) and the Universidad Peruana Cayetano Heredia (Perú). PERITAS project aims to (i) conduct abdominal US surveys to assess the prevalence of abdominal CE and identify clusters of infection in the all-age population of selected areas in Argentina, Chile and Peru; (ii) carry out environmental sampling for the detection of *E*. *granulosus* s.l. eggs; (iii) identify the potential risk factors associated with the transmission of *E*. *granulosus* s.l. to humans; and (iv) provide capacity-building training on infection prevention and the use of abdominal US for the diagnosis and management of CE to the medical personnel of target endemic areas.

Here, we present the results of the PERITAS project activities aimed to estimate by US screening the prevalence and the characteristics of human abdominal CE, and to identify the risk factors associated with human infection in rural and urban localities of the Monte Patria municipality, Limarí province, Coquimbo region, Chile.

## 2. Methods

### 2.1 Ethics statement

Approval was granted by the Scientific Ethics Committee of the Facultad de Medicina of the Universidad Católica del Norte, Coquimbo region, Chile (Resolución CECFAMED-UCN N° 81/2019). All participants were asked to sign a written informed consent and parental/guardian consent for the inclusion of those aged 5 to 17 years, and oral assent from children.

### 2.2 Study areas

**Fig 1:**
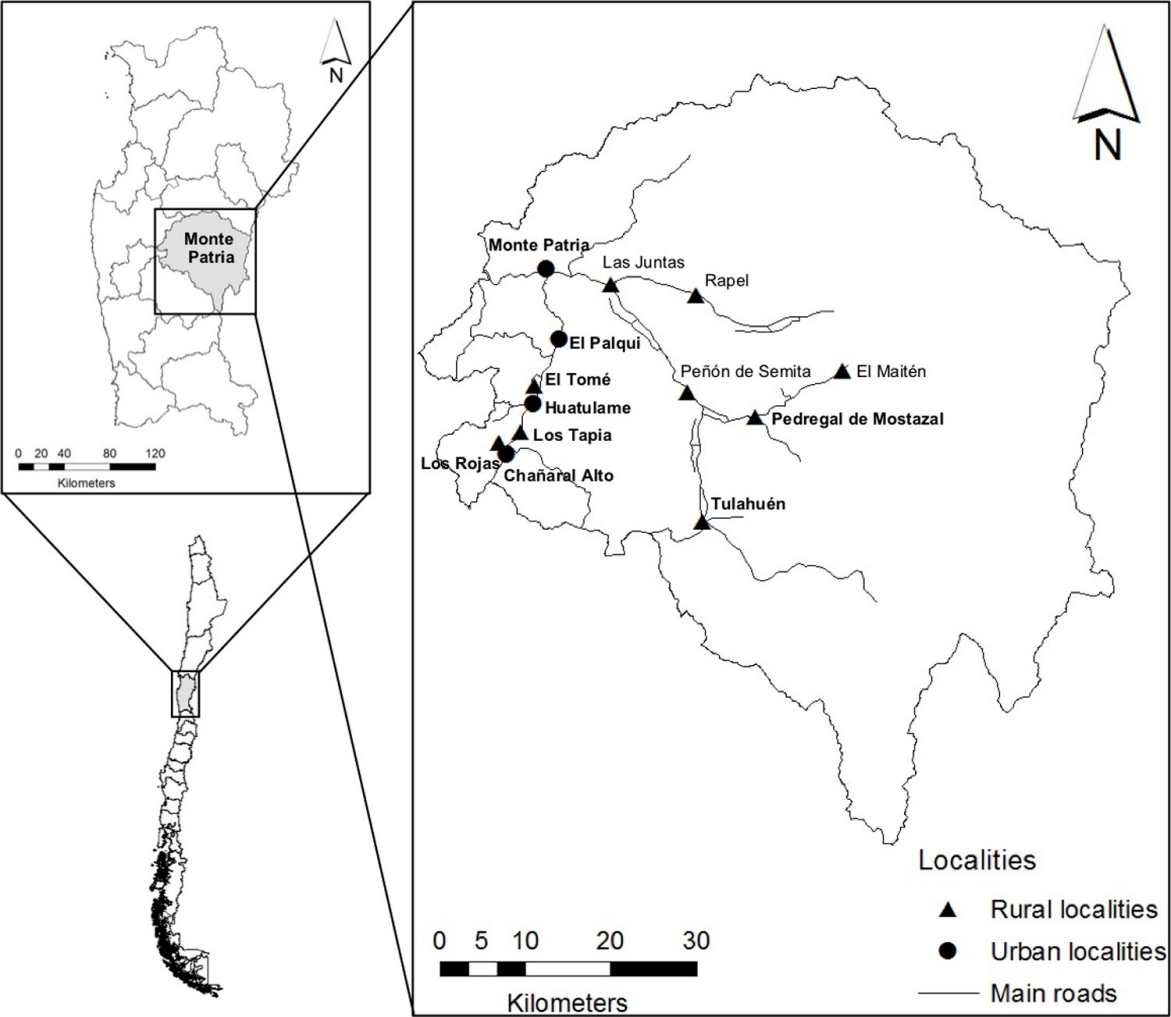
Map of localities of residence of volunteers screened by abdominal ultrasound in the Monte Patria municipality, Limarí province, Coquimbo region, Chile. Locality names in bold indicate the places where the ultrasound screening hubs were set-up in the study area. (Map made in QGIS Geographic Information System. Open Source Geospatial Foundation Project. http://qgis.osgeo.org. Shapes downloaded from an open source from the Biblioteca del Congreso Nacional, Available at https://www.bcn.cl/siit/mapas_vectoriales/index_html).

The study was conducted in selected rural and urban localities of the municipality of Monte Patria, Limarí province, Coquimbo region, Chile (Fig 1). Monte Patria covers an area of 4,366 km^2^ and is located at an altitude of 383 m.a.s.l., being 114 km distant from the closest major city (La Serena, capital of the region). Its population is estimated at 30,751 inhabitants, with a population density of 6.9 inhabitants/km^2^. People belonging to 13 localities were investigated, corresponding to four urban (Monte Patria: 6,533 inhabitants; El Palqui: 6,175; Chañaral Alto: 2,566; and Huatulame: 1,046) and nine rural (Tulahuén: 935 inhabitants; El Tomé: 535; Las Juntas: 399; Rapel: 336; Los Rojas: 310; Los Tapia: 300; Pedregal de Mostazal: 246; El Maitén: 76; and Peñón de Semita: 34) settlements (data retrieved from the Instituto Nacional de Estadísticas [23]) (Fig 1). These localities were primarily selected based on available human and animal data on the presence of CE; crucial factors for the maintenance of *E*. *granulosus* s.l. life cycle have been historically present throughout rural and urban areas of the province, such as home slaughter, feeding dogs with viscera and irregular/inadequate deworming of dogs [17,18]. For logistical constraints, the abdominal US survey was physically hosted in infrastructures of nine selected localities (Monte Patria, El Palqui, Chañaral Alto, Huatulame, Tulahuén, El Tomé, Los Rojas, Los Tapia and Pedregal de Mostazal), and people resident in the remaining four localities were mobilized to reach these study sites to be screened with US (Fig 1).

### 2.3 Ultrasound survey in urban and rural populations

The cross-sectional, community-based, abdominal US study was conducted between June and July 2019 on volunteers willing to participate who were residing in the selected communities, following coordination with local health authorities and community leaders. Pre-screening activities included informative talks about preventive measures of CE during community meetings. Regular announcements inviting residents to participate in the screening were made through local and regional newspapers, radio stations, handouts and municipal social networks. A 16-h lecture/hands-on US training based on the Focused Assessment with Sonography for Echinococcosis (FASE) protocol [24], was carried out targeting rural medical personnel of the Coquimbo region. The training was conducted in the municipality of Monte Patria by three researchers taking part in this study (LU, JCS, FT). None of the attending physicians had ever received training on CE and abdominal US before this project.

**Fig 2:**
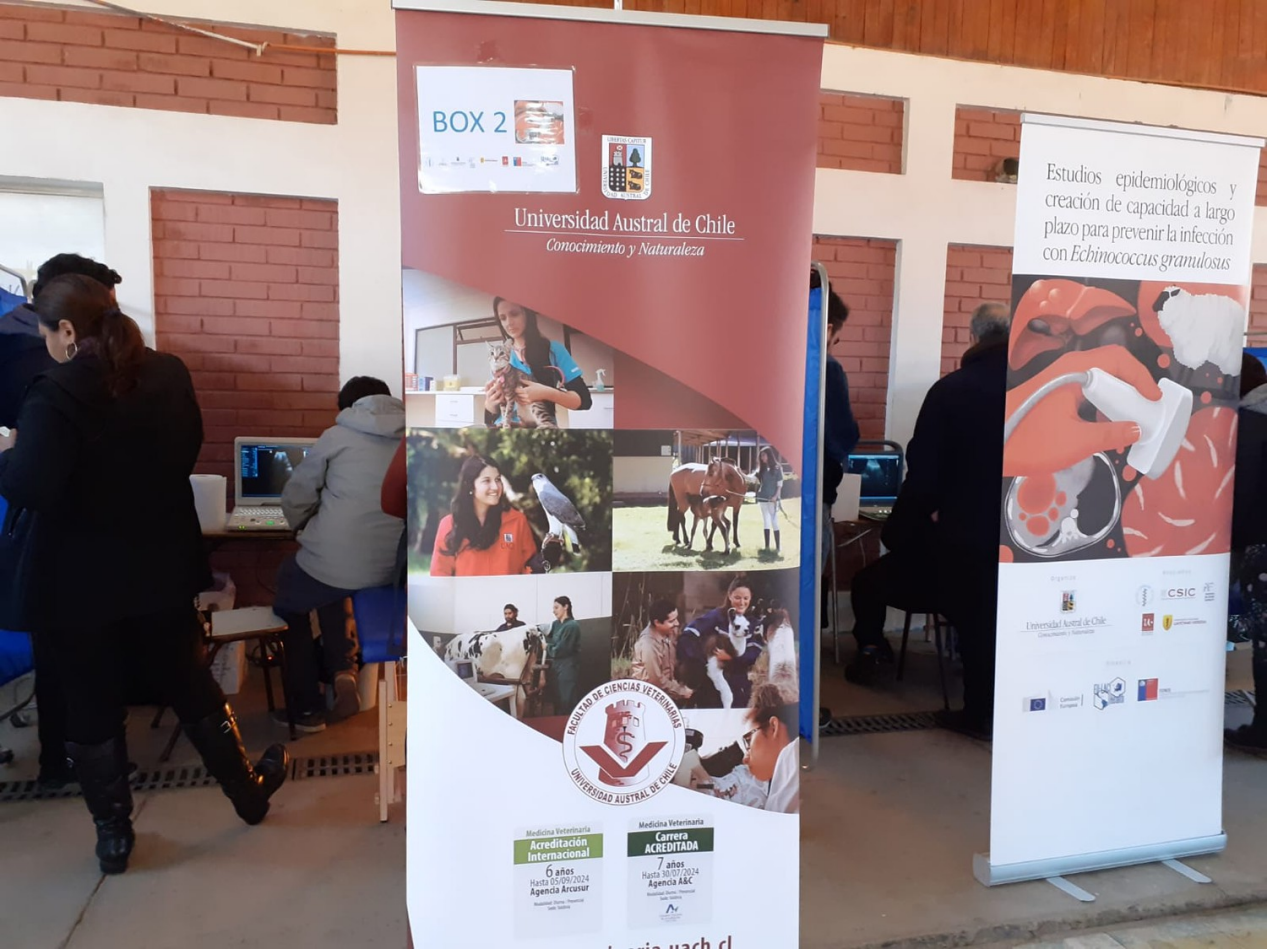
Example of a US-hub where abdominal US screening sessions were carried out in the study area.

Abdominal US screening sessions were hosted in public community structures such as community halls, schools and gyms that were distributed along nine of the target localities (Fig 2). Volunteers of both sexes willing to participate in the US survey were eligible if aged five years or older, and living in the target localities. Before being examined by US, all participants were informed about the US survey procedures and aims. Finally, all were asked about previous CE diagnosis and treatment.

The abdominal US examination, CE diagnosis and staging were performed according to the FASE protocol [24] and WHO Informal Working Group on Echinococcosis (WHO-IWGE) Expert Consensus [25] with three portable US devices (Sonoscape S2, Mindray Z6 and SonoSite M-Turbo, all with convex probe). For the study purposes, a CE case was defined by the visualization of pathognomonic features of CE on US imaging. A suspect CE case was defined as the presence of cysts without pathognomonic imaging features. Following US examination, all volunteers were provided with a complete written report that described the main US findings. The suggested clinical management was indicated for those individuals diagnosed with CE, as well as further diagnostic requirement for suspect cases [25]. All US screening procedures were conducted by three researchers taking part in this study (FT, LU, JCS) and were supported by regional physicians and field team operators.

### 2.4 Risk factor questionnaire

A standardized questionnaire about potential risk factors for human infection was answered by all participants before US examination (S1 Text). The information collected included demographic data (age, sex, occupation, locality of residence, time lived in the locality), dog tenure (dog ownership during the last five years), and implementation and frequency of personal behaviors possibly associated with the ingestion of *E*. *granulosus* s.l. eggs (touching dogs, eating own grown and raw unwashed vegetables, habits related to nail biting, smoking, use of toothpick or chewing tobacco/grass, hand washing before cooking/eating, and source of drinking water). The question on dog tenure was restricted to ownership in the past 5 years to limit recall bias also considering that, in the area, this habit is rather constant over time.

### 2.5 Analysis of data

All statistical analyses were performed using R v. 4.1.0 software [26]. CE prevalence values with 95% confidence interval (CI) were calculated according to total infection status and infection per each risk factor category. Univariate logistic regression models were used to assess the effect of demographic and behavioral factors on the odds of infection (CE-positive = 1, CE negative = 0). Variables were categorized as follows: 1) locality of residence (urban or rural); 2) time lived in the current locality (<5 and >5 years); 3) sex (male or female); 4) occupation (agricultural/livestock farming and non-agricultural/livestock farming); 5) dog ownership during the last five years (yes or no); and 6) each investigated behavioral habit (yes–and frequency of implementation–or no). Age was included as a continuous predictor in the models. The behaviors of hand washing before cooking and eating were not included in the analysis because all CE-cases stated that they always practiced the habit. A multivariate logistic regression model was constructed, starting with the inclusion of all factors that had a p-value ≤0.05 in the univariate models. For model selection, all the models were computed and ranked by AIC criteria corrected for small sample size (AICc) using the R-package MuMIn [27]. Prior to their inclusion in the models, predictor variables were tested for collinearity by assessing whether they affected the logistic regression coefficients of the other variables included in the models [28]. For all predictors in the univariate models and the best-ranked AIC multivariate model, odds ratios were calculated by exponentiating the logistic regression coefficients, and statistical significance was determined as a 95% CI that did not include the value 1.

## 3. Results

### 3.1 Demographic features of the target population

A total of 2,439 (~8% of municipality inhabitants) volunteers were examined by abdominal US; 1,678 (68.8%) came from urban areas and 761 (31.2%) from rural localities. The majority of screened volunteers (84.5%) reported being living in the corresponding locality for more than 5 years. The volunteers were aged 5–94 years, with 40±22 (mean ±SD) years of age in urban and 46 ±21 years of age in rural localities. The majority of volunteers were females in both urban (1,105/1,678; 65.9%) and rural (446/761; 58.6%) localities. Up to 30% of volunteers from rural areas worked in agricultural labors. The demographic features and behavioral characteristics that were investigated as potential risk factors for infection in the investigated sample are detailed in Table 1.

**Table 1:** Demographic and investigated behavioral features that could be risk factors for abdominal cystic echinococcosis in localities of the Monte Patria municipality, Chile.

	Locality	
	Urban	Rural
Total screened individuals	1,678	761
>5 years living in the current locality	82.0%	90.0%
Mean (±SD) age	40 (±22) years	46 (±21) years
Female sex	65.9%	58.6%
Working in agriculture or livestock farming	15.6%	30.4%
Dog ownership during last five years	74.3%	83.2%
Touching dogs	80.1%	79.1%
Eat self-grown vegetables	27.9%	36.4%
Eat raw unwashed vegetables	8.3%	3.3%
Nail biting	39.3%	31.1%
Smoking	16.8%	18.8%
Use toothpicks or chew grass blade/tobacco	22.4%	24.4%
Hand washing before cooking	96.4%	95.1%
Hand washing before eating	98.6%	99.5%
Drinking bottled water	66.7%	63.9%
Drinking private tap water	95.6%	96.5%
Drinking public tap water	27.0%	36.9%
Drinking non-potable water	19.8%	31.3%

### 3.2 Prevalence of CE and risk factor analyses

**Fig 3:**
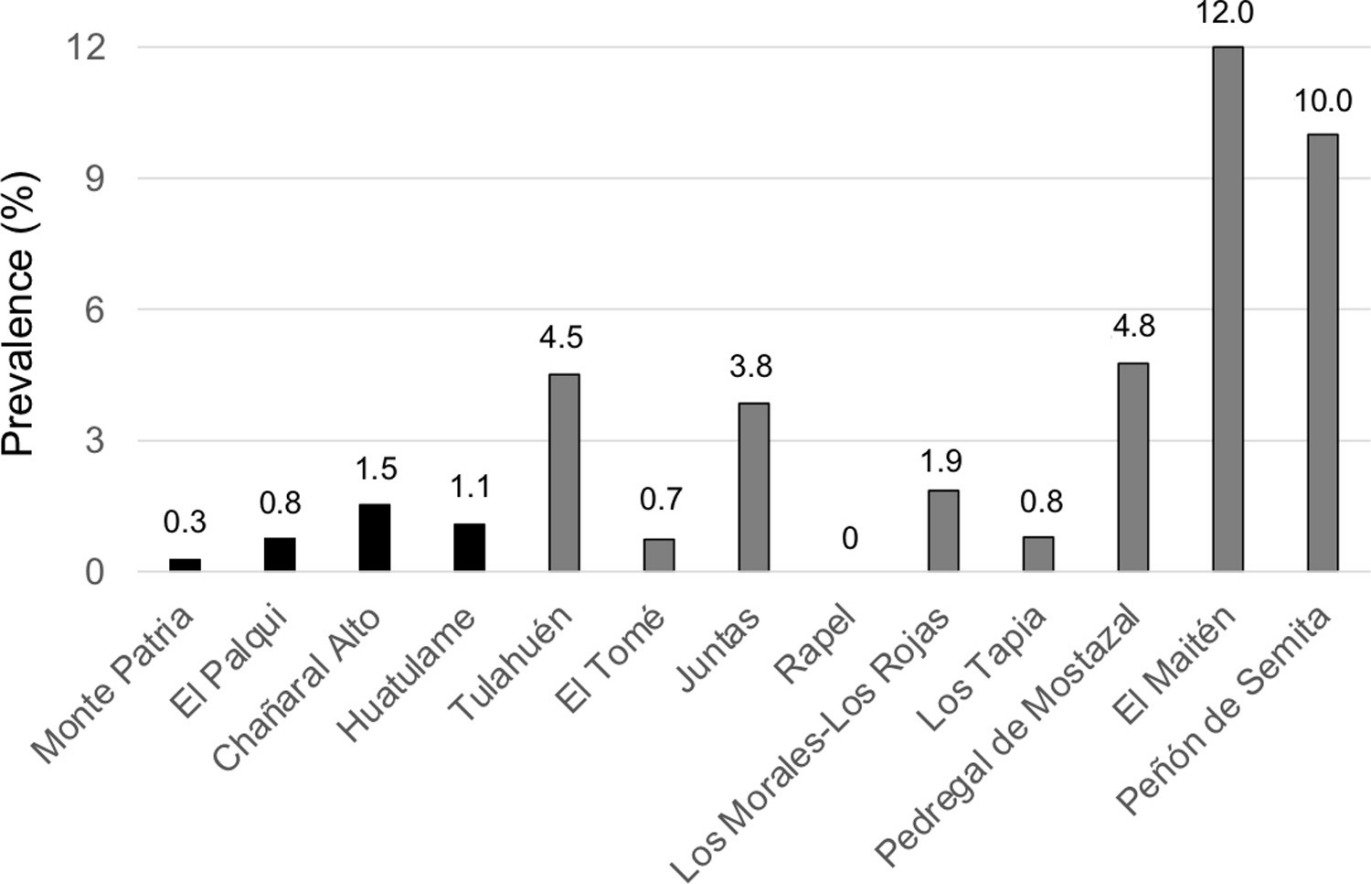
Prevalence of abdominal cystic echinococcosis according to the locality of residence in the Monte Patria municipality, Limarí province, Coquimbo region, Chile.

Of the 2,439 people who received abdominal US examination, 39 (1.6%; 95% CI 1.1–2.2) had at least one abdominal CE cyst. Volunteers with echinococcal infection, 17 (43.6%) males and 22 (56.4%) females, had a mean (±SD) age of 55.5 (±20) years (range 11–89 years). Across locality of residence, CE prevalence reached up to 12% (3/25; 95% CI 2.5–31.2) in rural localities (El Maitén) and 1.5% (6/393; 95% CI 0.6–3.3) in urban localities (Chañaral Alto) (Fig 3).

**Fig 4:**
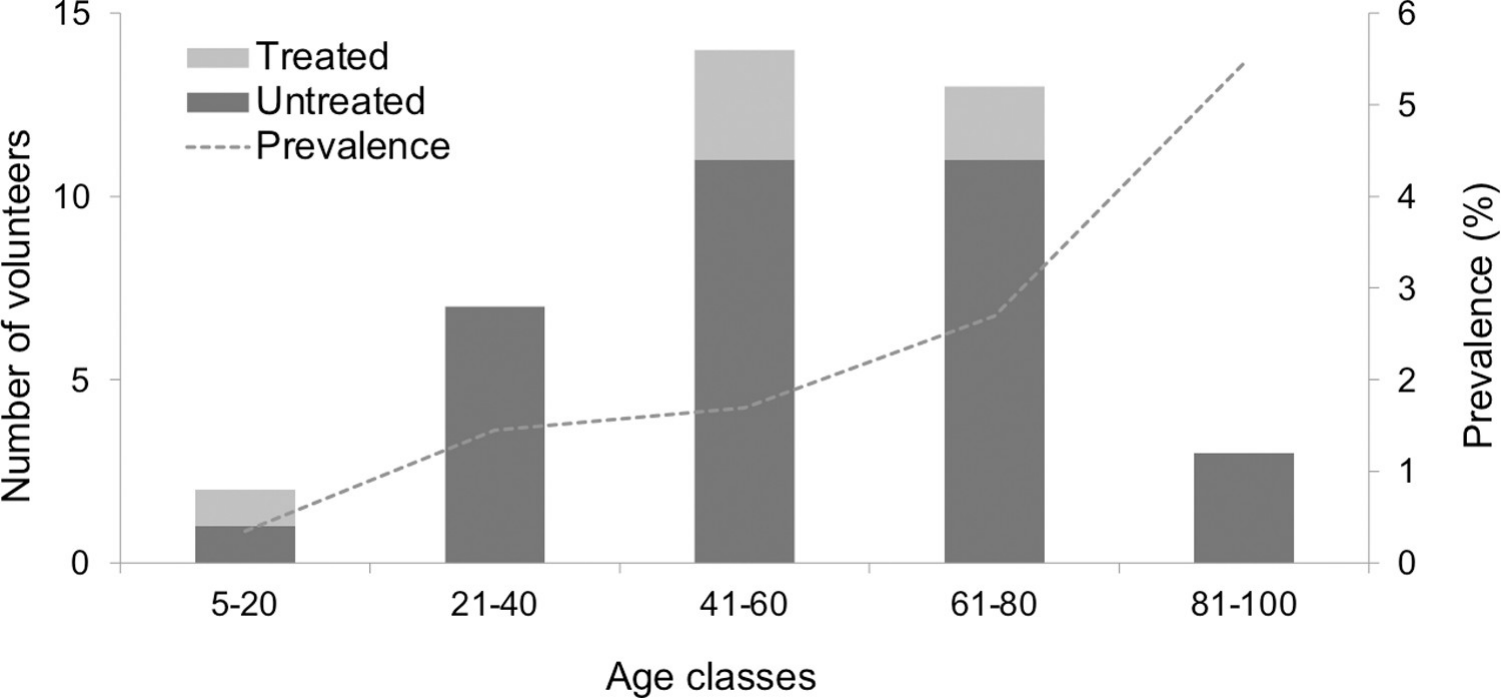
Distribution of abdominal cystic echinococcosis (CE) by age classes and prevalence. Number of volunteers with untreated abdominal CE (including new diagnoses and known but untreated infection) and volunteers with abdominal CE cysts on US who reported previous treatment for an abdominal CE cyst in any body location (bars; left Y axis) and overall prevalence of abdominal CE in the screened population (dotted line; right Y axis) according to age class (X axis). Age classes are expressed in years.

As shown in Table 2, the demographic and behavioral risk factors significantly related to human infection in the univariate logistic regression models were living in a rural locality, older age, working in agricultural/livestock farming activities, avoiding nail biting and frequently drinking non-potable water. Twenty-four out of 761 (3.2%; 95% CI 2.0–4.7) volunteers were found infected in rural localities, while 15 out 1,678 (0.9%; 95% CI 0.5–1.5) were found infected in urban localities. The odds of infection were over three times higher (OR = 3.61) for rural residents than for urban residents (Table 2). CE prevalence varied from 0.8% (9/1,060; 95% CI 0.4–1.6) in subjects aged 5–40 years to 2.2% (30/1,364; 95% CI 1.5–3.1) in volunteers aged >40 years (Fig 4). CE prevalence showed a statistically significant increase with age, odds of infection increasing by 3% per each year of age (OR = 1.03) (Table 2). Based on occupation, CE prevalence ranged from 1.3% (24/1,820; 95% CI 0.8–2.0) in non-agricultural/livestock workers (housewife, retired or other) to 2.8% (14/493; 95% CI 1.6–4.7) in agricultural/livestock workers. Odds of infection were over two times higher (OR = 2.19) for agricultural/livestock workers than for non-agricultural/livestock workers (Table 2). Regarding behavioral patterns reported by the volunteers, CE prevalence was 2.3% (9/386; 95% CI 1.2–4.4) in subjects who declared to rarely drink non-piped water and 4.3% (8/184; 95% CI 2.2–8.3) in subjects who declared frequently drinking non-potable water. Odds of infection were over three times higher (OR = 3.81) for people frequently drinking non-potable water than for people who did not report this behavior (Table 2). None of the remaining variables (time lived in the current locality, sex, dog ownership in the last five years, attitudes to touching dogs, eating self-grown/unwashed/uncooked vegetables, smoking, using toothpicks or chewing grass blades or tobacco, and drinking bottled, private or public tap water) were significantly related to infection in the univariate logistic regression models.

**Table 2:** Univariate risk factors for abdominal cystic echinococcosis in screened subjects from localities of the Monte Patria municipality, Limarí province, Coquimbo region, Chile.

Parameter	*n*	CE+	CE-	%	OR (95%CI_OR_)	*p*
*Locality of residence*						
Urban	1,678	15	1,663	0.9	1	
Rural	761	24	737	3.2	3.61 (1.90–7.07)	<0.01
*Years lived in the current locality*						
<5	151	2	149	1.3	1	
>5	2,061	35	2,026	1.7	1.29 (0.39–7.97)	0.73
*Age classes (years)*	2,424	39	2,385	1.6	1.03 (1.02–1.05)	<0.01
*Sex*						
Male	888	17	871	1.9	1	
Female	1,551	22	1,529	1.4	0.74 (0.39–1.42)	0.35
*Occupation*						
Housewife, retired or other	1,820	24	1,796	1.3	1	
Agricultural/livestock worker	493	14	479	2.8	2.19 (1.10–4.21)	0.02
*Dog ownership (last five years)*						
No	559	10	549	1.8	1	
Yes	1,879	29	1,850	1.5	0.86 (0.43–1.87)	0.69
*Touching dogs*						
Never	492	8	484	1.6	1	
Rarely	1,075	17	1,058	1.6	0.97 (0.43–2.40)	0.95
Frequently	871	14	857	1.6	0.99 (0.42–2.49)	0.98
*Eat grown vegetables*						
Never	1,693	28	1,665	1.7	1	
Rarely	550	8	542	1.5	0.88 (0.37–1.85)	0.75
Frequently	195	3	192	1.5	0.93 (0.22–2.65)	0.90
*Eat raw unwashed vegetables*						
Never	2,275	35	2,240	1.5	1	
Rarely	58	2	56	3.4	2.29 (0.37–7.75)	0.26
Frequently	105	2	103	1.9	1.24 (0.20–4.15)	0.77
*Nail biting*						
No	1,541	32	1,509	2.1	1	
Yes	897	7	890	0.8	0.37 (0.15–0.80)	0.02
*Smoking*						
No	2,013	36	1,977	1.8	1	
Yes	425	3	422	0.7	0.39 (0.09–1.09)	0.12
*Use toothpicks or chew grass/tobacco*						
No	1,876	31	1,845	1.7	1	
Yes	562	8	554	1.4	0.86 (0.37–1.79)	0.71
*Drinking bottled water*						
Never	832	12	820	1.4	1	
Rarely	1,247	23	1,224	1.8	1.28 (0.65–2.68)	0.49
Frequently	359	4	355	1.1	0.77 (0.21–2.23)	0.65
*Drinking private tap water*						
Never	100	1	99	1.0	1	
Rarely	214	3	211	1.4	1.41 (0.18–28.65)	0.77
Frequently	2,124	35	2,089	1.6	1.66 (0.35–29.62)	0.62
*Drinking public tap water*						
Never	1,704	31	1,673	1.8	1	
Rarely	335	3	332	0.9	0.49 (0.12–1.38)	0.24
Frequently	399	5	394	1.3	0.68 (0.23–1.62)	0.44
*Drinking non-potable water*						
Never	1,868	22	1,846	1.2	1	
Rarely	386	9	377	2.3	2.00 (0.87–4.25)	0.08
Frequently	184	8	176	4.3	3.81 (1.57–8.37)	<0.01

To increase the statistical power of the multivariate analysis, we combined the responses “rarely” or “frequently” *drinking non-potable water* as “yes” to produce two new categories, “yes” or “no”. The best-ranked AIC multivariate logistic regression model predicting human infection included the variables locality of residence, age class and drinking non-potable water (as binary category) (Table 3). Although volunteers that declared avoiding nail biting exhibited a higher likelihood of being infected than subjects that practiced nail biting, the inclusion of this factor evidenced collinearity with the variable age (nearly 10% variation in the logistic regression coefficient of age); thus, nail biting was not included in the multivariate modelling.

**Table 3:** Multivariate logistic regression model of risk factors for abdominal cystic echinococcosis in screened subjects from localities of the Monte Patria municipality, Limarí province, Coquimbo region, Chile.

Parameter	OR (95%CI_OR_)	*p*
*Locality of residence*		
Urban	1	
Rural	2.81 (1.46–5.56)	<0.01
*Drinking non-potable water*		
No	1	
Yes	2.16 (1.11–4.12)	0.02
*Age classes (years)*	1.03 (1.01–1.05)	<0.01

### 3.3 CE cysts features

All 39 subjects with abdominal CE cysts were asymptomatic, except one person who reported abdominal pain. Thirty-three infected volunteers (84.6%) declared that they had not been diagnosed or treated for CE before, they were therefore newly diagnosed with this parasitic infection during the study screening; while thirty individuals (76.9%) aged over 40 years, declared having been previously treated for CE (Fig 4). The volunteers having declared previous treatment for CE reported having had cysts in the liver (*n* = 4), lungs (*n* = 1) and both liver and lungs (*n* = 1); one subject was treated surgically, two received medical treatment with albendazole, two received both surgical and medical treatment, and one percutaneous treatment. No residual lesions from previous surgery for abdominal CE were found during the US screening. Other incidental findings in 16.8% (410/2,439) volunteers were vesicular lithiasis (*n* = 198), simple biliary cysts (*n* = 56), kidney lesions (*n* = 73), prostatic hypertrophy (*n* = 56), and other miscellaneous findings (*n* = 27).

**Fig 5:**
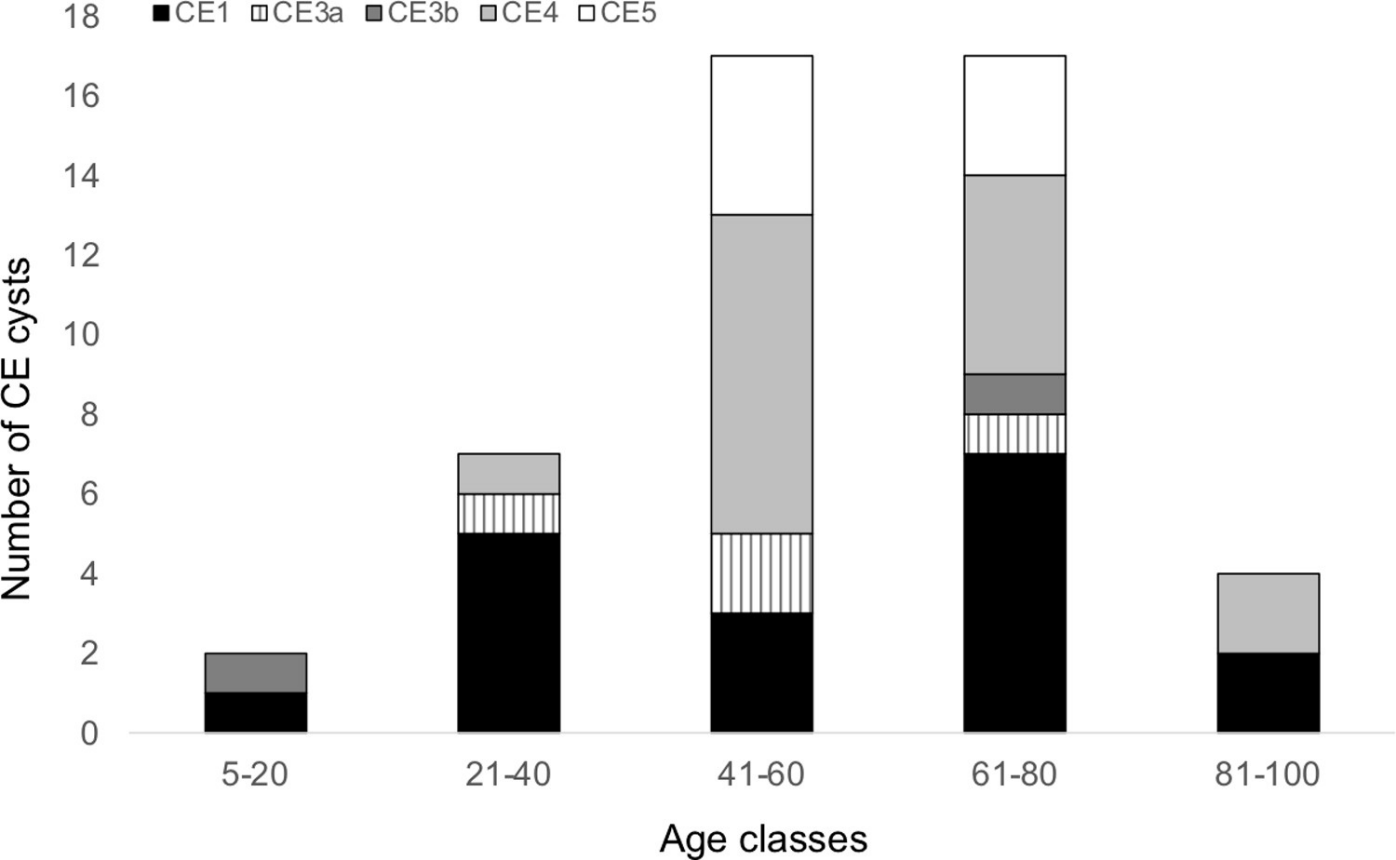
Distribution of the abdominal cystic echinococcosis cyst stages (47 cysts detected in 39 cases) according to the WHO Informal Working Group on Echinococcosis (WHO-IWGE) classification by age classes. Monte Patria municipality, Limarí province, Coquimbo region, Chile.

The 39 volunteers with abdominal CE had a total of 47 CE cysts (mean 1.2 CE cysts per subject; range: 1–3). Thirty-seven of these subjects (94.9%) had CE cysts in the liver, while two (5.1%) had cysts in the central abdomen and left kidney, respectively. According to the WHO-IWGE classification, 24 (51.1%) cysts were in active stages (18 CE1 [unilocular fluid-filled with double-wall sign], 4 CE3a [with detached parasitic layers], and 2 CE3b [solid content with folded parasitic membranes and daughter cysts]), while 23 (48.9%) cysts were in inactive CE4 (with solid content and folded parasitic layers) or CE5 (CE4 features and calcified walls) stage. Cysts in the CE1 stage were detected in volunteers throughout all age classes (11–89 years). Twelve (66.7%) of cysts in the CE1 stage and 22 (95.7%) of cysts in the CE4/CE5 stages were detected in volunteers aged >40 years (Fig 5). Of the nine <21 years-old infected subjects (which included two minors aged 11 and 17 years), eight had cysts in active stages (CE1, CE3a or CE3b), and one had an inactive CE4 cyst. Of the six previously treated volunteers, two (33%) still had active cysts (CE3b stage) on US, while the other four (67%) had inactive cysts (CE4 or CE5 stages). Due to a lack of medical records, it was not possible to discriminate between new infections and CE cysts still present post-treatment.

## Discussion

In this study, conducted through a European-South American partnership, we estimated the prevalence and the characteristics of human abdominal CE by means of a community-based US screening, and identified a number of potential risk factors associated with infection in rural and urban localities of the Monte Patria municipality (Limarí province, Coquimbo region, Chile). We detected an overall prevalence of human CE of 1.6% across the sampled localities and infection was significantly associated with rural residence, older age and drinking non-piped water.

Of the subjects with CE, 84.6% were newly diagnosed. Active cysts were detected in all age classes (including children and subjects >80 years of age), while 95.7% of inactive cysts were found in >40 years-old subjects. Different CE prevalence rates were found between localities of residence, the likelihood of infection being significantly higher in rural (3.2%) than urban (0.9%) localities. These findings are in accordance with other studies conducted in Chile [16–18] and elsewhere [6,29,30], supporting the evidence that transmission of this zoonotic parasitosis is strongly linked with residence in a rural environment, plausibly more contaminated by parasite eggs, to which people are cumulatively exposed over time. In the Limarí province, rural economy has been historically based on small ruminant breeding [18], and at-risk practices such as home livestock slaughter and feeding of dogs with raw viscera cause dog infection and consequently shedding of infected feces, perpetuating the *E*. *granulosus* s.l. infection cycle and the contamination of the environment [15,17]. Indeed, the significant increase of CE prevalence with age (0.8% in 5–40 years-old subjects versus 2.2% in >40 years-old subjects), together with the observation of active cysts, arguably reflecting more recent infection in all age groups, reflects what is expected for an environmentally transmitted infection, where older people have increasing opportunities to come into contact with eggs-contaminated matrices across their lifetime [6,22,30]. Interestingly, agricultural/livestock occupation was significantly associated with an increased odds of infection in the univariate analysis, but not retained in the final multivariate model. One third of the inhabitants of the sampled rural localities stated that they worked as farmers, livestock breeders or conducted other agricultural activities, and thus, they could be susceptible to acquiring the infection through persisting opportunities of contact with egg-contaminated matrices within rural environments [29].

Water drinking habits was the only specific behavior supported as significant predictor of infection in the final model, while specific hand-to-mouth habits were not. Nail biting, negatively correlated with infection in the univariate analysis, showed collinearity with age and was therefore excluded from the multivariate model. Again, these results are in line with the hypothesis that “cumulative” exposure over time to *E*. *granulosus* s.l. eggs in a contaminated environment (i.e. matrices in general) is the main risk factor for infection, while occasions of eggs ingestions are difficult to individuate, even when investigating specific habits and their frequency of implementation, like in this study. The non-potable water frequently drunk by about 30% of the studied rural residents may have increased their contact with infective eggs of *E*. *granulosus* s.l., resulting in a higher infection likelihood in the screened volunteers. This behavior has also been described as a significant risk factor for CE in Asian countries, such as Jordan [31], Kyrgyzstan [32], China [33], and South American countries, such as Perú [34], Uruguay [35,36] and Argentina [30,37], but the same association was found inconsistently in Bulgaria, Romania and Turkey [29] and non-univocal results were found by studies reviewing risk factors for CE at global level [6,7]. Clearly, different risk factors and environmental conditions may be predominant in different areas [8]. It must also be considered that there is a degree of recall bias and reporting bias caused by the knowledge that some habits are unhealthy, inducing denial of the actual actuation, a burden on questionnaire-based results.

Despite the key epidemiological role of dogs in the *E*. *granulosus* s.l. life cycle, owned dog-related factors (owning dogs and length of dog ownership in the last 5 years) and physical contact with the definitive host (frequently touching dogs, irrespective of their ownership), were not found associated with odds of human infection in this study. Again, this result is in line with some and in contrast with other previous studies [6], although comparisons of results are difficult to make due to the heterogeneity of meaning which could have been applied in different circumstances and cultures for the concepts of “dog ownership” and “touching dogs”. It is plausible that environmental contamination with parasite eggs by infected roaming dog feces occur across the entire community regardless of the dog ownership declared by the interviewed person, as also discussed in previous studies [19,29]. To better understand and model the pathways of transmission and source attributable fractions of human infection in specific areas, an integrated approach that encompasses both specifically designed questionnaires and molecular-epidemiological studies sampling different matrices for the presence of viable parasitic eggs is needed [7,8].

Across all subjects diagnosed with CE by US screening, we found significantly more previously unknown and/or untreated infections (*n* = 33) than treated infections (*n* = 6). Almost all the former individuals were asymptomatic. While this lack of symptoms can persist as such even lifelong, sudden and life-threatening complications of the infection, such as rupture, can occur and highlight the value of population screening and early diagnosis of infection followed by appropriate, stage-specific clinical management. We detected a fairly similar percentage of active (51.1%) and inactive (48.9%) CE cysts, likely reflecting the presence of both recent and chronic infections in the screened population [38,39]. Adult volunteers over 40 years of age had the highest percentage of inactive CE4/CE5 cyst stages (95.7%), which aligns to findings of previous longitudinal and observational studies evidencing that a considerable proportion of cysts evolve spontaneously to inactivation over time [19,38,40–42]. The detection of a relatively high percentage of active CE1 cyst stages in people of all age groups may be interpreted as the infection could be acquired at all ages or the infection is acquired in youth and the cyst persists stable in CE1 stage for many decades. Unfortunately, it is very difficult to discern between these two instances, unless observational longitudinal studies in the all-age population are performed, ideally (but ethically arguable) in the absence of treatment.

Active surveillance studies by US survey aimed to detect infections not reaching medical attention have been carried out in many countries, some of which were previously cited. This is the first US survey in Chile and our findings suggest that local prevalence of CE has been largely underestimated. Newly diagnosed infections (either recent or old infections) in untreated children and adults represent a public health issue in rural and urban localities of the Limarí province. Factors related to a higher risk of CE found in this study suggest that people are infected while residing in rural localities (where environmental contamination most likely occurs), older age groups are more “cumulatively” exposed to the parasitic infection, and drinking non-potable water may increase the likelihood of infection across the target localities. The identification of these factors may facilitate the implementation of geographically-focused, cost-effective diagnostic and control strategies and the improvement of the epidemiological surveillance system of the region and in Chile.

This study had several limitations and potential sources of bias which could have influenced the results in either direction, with under- or over-estimation of CE prevalence. Firstly, the non-random selection of participants could have affected our prevalence estimations but is largely applied in similar population-based studies for CE for both ethical, social, and practical reasons [22]. Difficulties in people mobilization from remote rural localities to US hubs and, conversely, the higher number of volunteers screened in urban areas, might have also contributed to selection bias. Individuals already diagnosed and treated might have had an increased interest in attending the study for a free revision, but this was only recorded in six (15%) volunteers. One of the most important biases for our estimates is the low recruitment of individuals living in rural sites since, according to the national census, 54% of people live in rural areas in Monte Patria [23] but our sample consisted of only 31% of people from these areas, which could have underestimated the real prevalence of CE. The sex balance in the participants sample was slightly biased towards females (population of 50.5% and sample of 63.6% females) [23], as very common also in similar studies [eg. 6,30]. However, it is difficult to evaluate in what direction this could have influenced the estimation of prevalence, since it is difficult to discern whether, in each setting, one sex might be more exposed to infection than the other, and similar data in the literature are conflicting and prone to bias [6,30,43]. Regarding age, our sampling overrepresented ages above 45 years (approximately 12% more than the target general population) [23], which could have induced an overestimation of the prevalence. Finally, our results are limited by the relatively small sample size, which could have affected the possibility of detecting other risk factors. Further investigations are needed to increase the number of localities in other areas in Chile. In fact, PERITAS project was originally planning a second US survey in Patagonia, however COVID-19 pandemic and national lock-downs did not allow the implementation of the screening in this area. Further active surveillance by US surveys aimed to detect CE cases in Chile are strongly recommended.

## Supporting information

S1 Text Epidemiological questionnaire applied to volunteers before US examination. (DOCX)
